# MESSENGER Observations of Planetary Ion Enhancements at Mercury's Northern Magnetospheric Cusp During Flux Transfer Event Showers

**DOI:** 10.1029/2022JA030280

**Published:** 2022-04-15

**Authors:** Weijie Sun, James A. Slavin, Anna Milillo, Ryan M. Dewey, Stefano Orsini, Xianzhe Jia, Jim M. Raines, Stefano Livi, Jamie M. Jasinski, Suiyan Fu, Jiutong Zhao, Qiu‐Gang Zong, Yoshifumi Saito, Changkun Li

**Affiliations:** ^1^ Department of Climate and Space Sciences and Engineering University of Michigan Ann Arbor MI USA; ^2^ Institute of Space Astrophysics and Planetology INAF Rome Italy; ^3^ Southwest Research Institute San Antonio TX USA; ^4^ NASA Jet Propulsion Laboratory California Institute of Technology Pasadena CA USA; ^5^ School of Earth and Space Sciences Peking University Beijing China; ^6^ Japan Aerospace Exploration Agency Institute of Space and Astronautical Science Sagamihara Japan

**Keywords:** Mercury, solar wind‐magnetosphere‐surface coupling, solar wind sputtering, planetary ions enhancements, planetary ion escape rate

## Abstract

At Mercury, several processes can release ions and neutrals out of the planet's surface. Here we present enhancements of planetary ions (Na^+^‐group ions) in Mercury's northern magnetospheric cusp during flux transfer event (FTE) “showers.” FTE showers are intervals of intense dayside magnetopause reconnection, during which FTEs are observed in quick succession, that is, only separated by a few seconds. This study identifies 1953 FTE shower intervals and 1795 Non‐FTE shower intervals. During the shower intervals, this study shows that the FTEs form a solar wind entry layer equatorward of the northern magnetospheric cusp. In this entry layer, solar wind ions are accelerated and move downward (i.e., planetward) toward the cusp, which sputter upward‐moving planetary ions with a particle flux of 1 × 10^11^ m^−2^ s^−1^ within 1 min. The precipitation rate is estimated to increase by an order of magnitude during FTE showers, to 2 × 10^25^ s^−1^, and the neutral density of the exosphere could vary by >10% in response to this FTE‐driven sputtering. Such rapid large‐scale variations driven by dayside reconnection may explain the minute‐to‐minute changes in Mercury's exosphere, especially on the high latitudes, observed by ground‐based telescopes on Earth. Our MESSENGER in situ observation of enhanced planetary ions in the entry layer likely corresponds to an escape channel for Mercury's planetary ions. Comprehensive, future multipoint measurements made by BepiColombo will greatly enhance our understanding of the processes contributing to Mercury's dynamic exosphere and magnetosphere.

## Key Points

- Solar wind entry layer is observed to form equatorward of cusp during flux transfer event showers which enhances precipitation rate by an order of magnitude
- Solar wind sputtering can release planetary ions and neutrals efficiently within 1 min of the onset of magnetopause reconnection
- A new escape channel for planetary ions is formed by solar wind sputtering with an escape rate of ∼10^24^ s^−1^ for Na^+^‐group ions

## 1 Introduction

Mercury possesses a global dipole magnetic field with a similar polarity to Earth's dipole field, but the magnetic field intensity near Mercury's magnetic equatorial plane (∼200 nT) is much less than the strength of Earth's field (∼30,000 nT) (see Anderson et al., 2012). Mercury's magnetic field can hold off the constantly streaming solar wind with a subsolar magnetopause distance of around one thousand kilometers above the planet's surface (Siscoe et al., 1975; Slavin et al., 2008). As the closest planet to the Sun, Mercury is subject to the strongest solar wind driving compared to other planets in the solar system (Slavin & Holzer, 1981; Sun et al., 2022). One outcome is that the magnetic reconnection erosion effect on the dayside magnetopause is significant at Mercury (Slavin & Holzer, 1979; Slavin et al., 2014, 2019), and it often generates flux transfer events (FTEs) on the magnetopause (Russell & Elphic, 1978; Russell & Walker, 1985; Slavin et al., 2009, 2012; Sun, Slavin, Smith, et al., 2020). In Mercury's magnetosphere, the FTEs are observed often in quick succession and a large number by MErcury Surface, Space ENvironment, GEochemistry, and Ranging (MESSENGER, Solomon et al., 2007), which is named flux transfer event (FTE) “shower” (Slavin et al., 2012, 2014, 2019; Sun, Slavin, Smith, et al., 2020).

The occurrence of FTE showers is high and depends on both of the magnetosheath plasma β, which is the ratio of the thermal pressure to the magnetic pressure, and the magnetic shear angle across the magnetopause (Sun, Slavin, Smith, et al., 2020). The high occurrence of FTE showers at Mercury is due to Mercury's magnetosphere being embedded in the solar wind with a low Alfvénic Mach number (*M*_A_ ≲ 4) (Slavin & Holzer, 1981; Sun et al., 2022), that is, the ratio of solar wind speed to the Alfvén speed. The low *M*_A_ solar wind often leads to a magnetosheath with a low plasma β that forms a plasma depletion layer (PDL) ahead of the magnetopause (Gershman et al., 2013; Slavin et al., 2014). As a result, magnetic reconnection at Mercury's magnetopause would be less dependent on the polarity of the IMF and occurs at a high rate (Slavin et al., 2014; Sun, Slavin, Smith, et al., 2020). In addition, when reconnection occurs, the reconnecting rate is large (DiBraccio et al., 2013; Slavin et al., 2009). This can be revealed by the fact that not only do FTE showers occur in high occurrence rates (around 52% of the magnetopause crossings) but also the FTEs are observed in quick succession, that is, separated by only a few seconds. FTEs occur at lower rates and are separated by tens of minutes at other planets (Jasinski, Akhavan‐Tafti, et al., 2021; Russell, 1995), such as Earth, Jupiter, and Saturn since they are embedded in the solar wind with a higher *M*_A_, see Sun et al. (2022) for a review.

Mercury does not have a significant atmosphere but a tenuous exosphere. Since the discovery of Mercury's neutral exosphere (Broadfoot et al., 1974; Potter & Morgan, 1985), thermal and photon‐stimulated desorption (Madey et al., 1998; Sprague et al., 1997), micrometeoroid impact vaporization (Jasinski et al., 2020; Mangano et al., 2007; Morgan et al., 1988) and the solar wind sputtering have been proposed to release neutral particles (Hofer, 1991; Killen et al., 2001; McGrath et al., 1986; Mura et al., 2005) and ions (Benninghoven, 1975; Broadfoot et al., 1974; Raines et al., 2014) from the planet's surface. However, these processes and their relative importance are poorly understood. Magnetic field lines in the FTEs are open field lines with one end connecting to the solar wind and the other to the planetary magnetic field in the magnetospheric cusp (Lee & Fu, 1985). In these field lines, solar wind particles can be accelerated and transported into the magnetosphere and bombard the regolith beneath the cusps, which corresponds to the process of solar wind sputtering (Hofer, 1991; Killen et al., 2001; McGrath et al., 1986; Mura et al., 2005). The investigation of solar wind sputtering has been limited to theoretical models in Mercury's studies. There is still no in situ evidence for this process.

Here we present MESSENGER's observations of the enhancement of planetary ions, specifically of the Na^+^‐group ions, which includes Na^+^ (sodium ion), Mg^+^ (magnesium ion), Al^+^ (aluminum ion), and Si^+^ (silicon ion), near Mercury's northern magnetospheric cusp during flux transfer event (FTE) “showers.” The FTEs accelerate and magnetically channel solar wind protons downwards and planetwards toward the magnetospheric cusps, which forms a solar wind entry layer. This entry layer is observed to increase the proton precipitation rate beneath the cusps at the planet's surface by an order of magnitude. This produces antiplanetward‐moving planetary ions within around 1 min after the onset of an FTE shower. The neutral density of the exosphere can vary by >10% due to this FTE‐driven sputtering. These in situ observations of enhanced planetary ions in the entry layer likely correspond to an escape channel of Mercury's planetary ions, and the large‐scale variations of the exosphere observed on minute‐timescales by ground‐based solar telescopes.

## 2 Satellite and Instrumentation

MESSENGER orbited Mercury between 18 March 2011, and 30 April 2015, UTC, around 17 Mercury years. In this study, measurements of the magnetic field made by the Magnetometer (MAG) (Anderson et al., 2007) and of ions by the Fast Imaging Plasma Spectrometer (FIPS), which is a part of Energetic Particle and Plasma Spectrometer (EPPS) (Andrews et al., 2007), were used. The MAG provided the magnetic field at a time resolution of 20 vectors per second in the Mercury solar orbital (MSO) coordinate system.

The Fast Imaging Plasma Spectrometer (FIPS) was an ion‐mass spectrometer, which could resolve mass per charge from 1 to 60 amu/e through energy per charge (E/q) and time‐of‐flight (TOF) measurement. The range of energy per charge of FIPS was from about 0.1 to 13.5 keV/e with a time resolution of ∼10 s (scan time was ∼8 s). FIPS used the double‐coincidence technique, which greatly reduced background noise. FIPS had a field‐of‐view (FOV) of around 1.4 πsr and an angular resolution of 10°. However, the spacecraft obstructions reduced the FOV to an effective value of ∼1.15 πsr.

Sodium‐group, Na^+^‐group, ion data used in this study contained ions of mass per charge from 21 to 30 amu/e, which included Na^+^, Mg^+^, Al^+^, and Si^+^ (Raines et al., 2013). The measurements were binned to increase the signal‐to‐noise ratio, and it is not possible to directly distinguish between these species at present. The Na^+^‐group ions in this paper refer to the above‐mentioned group of species. The densities of Na^+^‐group ions and the Alpha particle (He^++^) used in this study were the observed number density. The observed density was calculated by integrating the phase‐space density, which is converted from the observed counts, over the observed velocity range and FOV. The real density should be about 3.48 (4 π/1.15 π) times the observed density. A detailed description of the FIPS, including its FOV, double‐coincidence technique, and derivation of observed density, was given by Raines et al. (2011, 2013).

The aberrated Mercury solar orbital (aMSO) coordinates were used for the spacecraft position. In the MSO coordinates, the ${\hat{x}}_{\text{MSO}}$ points from the center of Mercury to the Sun, the ${\hat{z}}_{\text{MSO}}$ points northward perpendicular to the Mercury's orbital plane, and the ${\hat{y}}_{\text{MSO}}$ completes the right‐handed system, which is directed opposite to Mercury's orbital velocity around the Sun. In the aMSO, the coordinates of ${\hat{x}}_{\text{aMSO}}$−${\hat{y}}_{\text{aMSO}}$ plane is rotated to be antiparallel to the solar wind (400 km/s along −${\hat{x}}_{\text{aMSO}}$).

The aberrated Mercury solar magnetospheric (aMSM) coordinates were used for the magnetic field data. Because the dipole axis of Mercury is close to the ${\hat{z}}_{\text{MSO}}$ with an angle difference of <0.8°, the three‐axis of aMSM are the same as those of aMSO except that the origin of aMSM is at the center of the dipole, which is shifted northward of approximately 484 km from the planetocentric position.

## 3 FTE Shower on 22 March 2012

### 3.1 Event Overview

**Figure 1:**
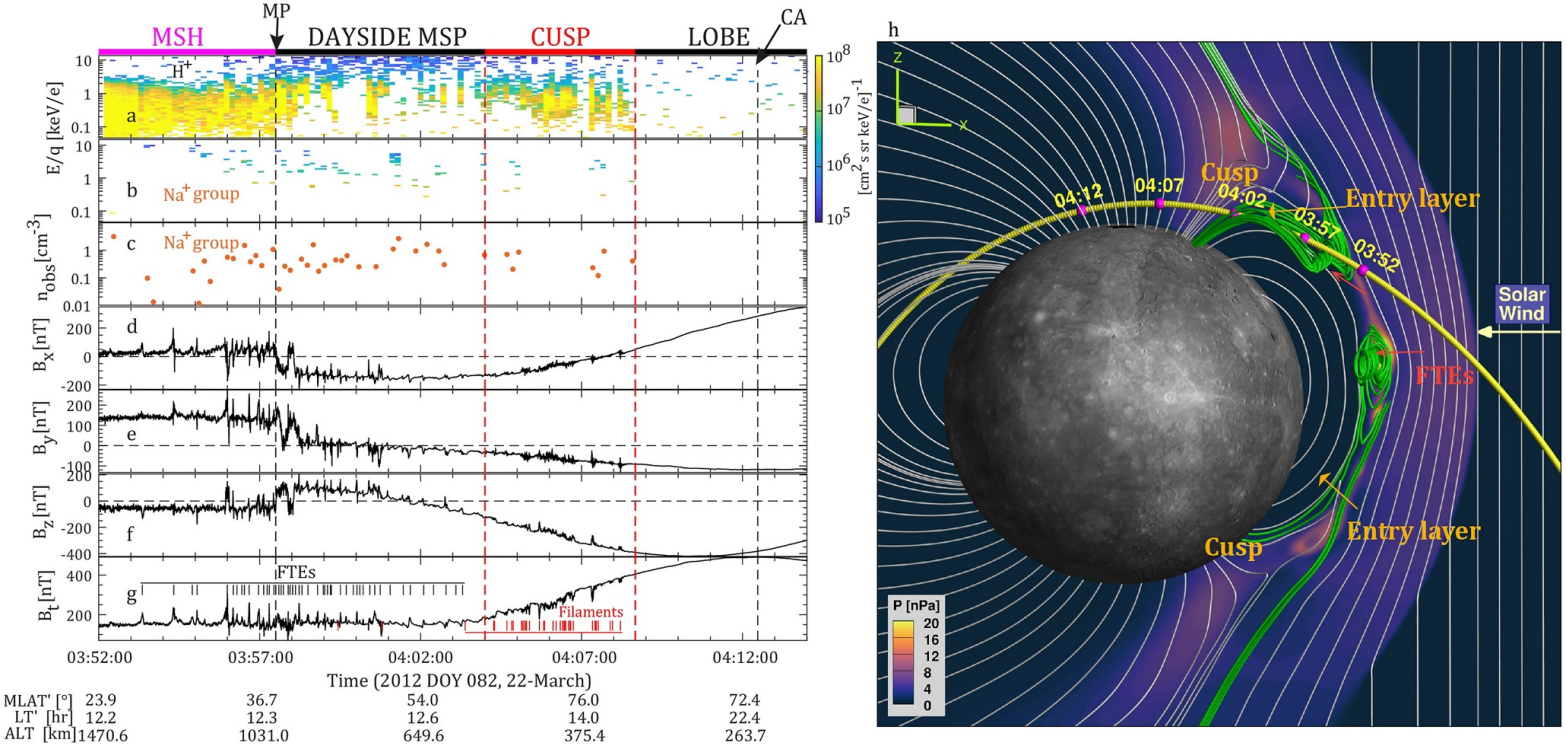
Overview of the flux transfer event (FTE) shower observed by MErcury Surface, Space ENvironment, GEochemistry, and Ranging (MESSENGER) on 22 March 2012 (left) and the Hall‐MHD simulation (right). Left: (a), differential particle flux for protons (see Section 2), (b) differential particle flux for sodium group ions (Na^+^‐group ions, *m*/*q* = 21–30), (c) observed densities of Na^+^‐group ions. (d–f) Magnetic field components, *B*_x_, *B*_y_, *B*_z_, respectively, (g) magnetic field intensity *B*_t_. The black and red ticks mark FTEs and cusp filaments, respectively. Observations in the magnetosheath (MSH), dayside magnetosphere (Dayside MSP), cusp and lobe are labeled at the top of the figure. Magnetic Latitude (MLAT), Local Time (LT), and Altitude (ALT) of the spacecraft are labeled underneath the figure. The dayside magnetopause (MP) crossing occurred at an altitude of ∼990 km, close to the equator (∼37.2°) and at local noon (∼12:20 local time). Closest approach (CA) occurred at an altitude of ∼263 km at high latitudes (∼71.0°) and at local midnight (∼22:35 LT). Right: (h) the BATSRUS Hall‐Magnetohydrodynamics (MHD) simulation of Mercury's magnetosphere under similar interplanetary magnetic field (IMF) conditions to the MESSENGER observations. In this snapshot, two FTEs with a clear helical magnetic field topology (green lines) appeared simultaneously at the dayside magnetopause. The solar wind entry layers were identified equatorward of the northern and southern cusps, respectively. The cusps are identified as the regions with higher thermal pressure. The yellow line represents MESSENGER's trajectory, which contains some time marks consistent with the left panel.

On the left side of Figure 1, the measurements of the magnetic field and ions during one of the MESSENGER's descending orbits on 22 March 2012 are displayed. In around 20 min, MESSENGER traveled from the subsolar magnetopause to the northern cusp and then reached the closest approach (CA) at an altitude of around 260 km, during which MESSENGER observed abundant Na^+^‐group ions (including Na^+^, Mg^+^, Al^+^, and Si^+^) with the observed densities (*n*_obs_) of 0.5–2 cm^−3^ (Figure 1c). Early in the time series, near the magnetopause, MESSENGER observed approximately 40 FTEs in less than 5 min. These FTEs, separated by only a few seconds, appeared in a large group, which corresponded to an interval of FTE shower.

During this event, the interplanetary magnetic field (IMF) in the magnetosheath was southward with a magnetic shear angle across the magnetopause of 107°. The magnetic field intensity in the magnetosheath was slightly smaller than the field intensity in the magnetosphere, which implied a plasma depletion layer (PDL) ahead of the magnetopause (Gershman et al., 2013), and the plasma β was calculated to be approximately 0.08 in the magnetosheath. As the plasma β in the dayside magnetosphere was small (<0.1), the magnetic reconnection was approximately symmetric at this magnetopause.

This event at Mercury's magnetosphere was modeled by a global Hall‐Magnetohydrodynamics (MHD) simulation with a coupled planetary interior (Chen et al., 2019; Jia et al., 2015, 2019) under similar low magnetosheath plasma β and southward IMF conditions. See Appendix A on the introduction of the Hall‐MHD simulation. On the right‐hand side of Figure 1, magnetic flux ropes centered in the FTEs with helical magnetic field lines are gathered on the dayside magnetopause. These flux ropes are formed between neighboring X‐lines, in which the magnetic field lines have one end in the solar wind while the other passes through the northern or southern cusps and down into Mercury's surface. Consequently, for the present case in the northern hemisphere, the solar wind particles parallel to the magnetic field lines would travel into the magnetosphere along the open magnetic field lines inside the FTEs. In Figure 1a, the intermittently appearing cold (≲1 keV) and dense protons in the high latitude magnetosphere correspond to these injected ions. These injected solar wind protons are further analyzed in the next section. In the lower altitude magnetosphere, including the cusp, the injected solar wind particles along the open field lines inside the FTEs diamagnetically reduce the planetary magnetic field and generate magnetic depressions, which are known as filaments (see Figure 1g and ref. Slavin et al., 2014; Poh et al., 2016).

### 3.2 Injected Solar Wind Protons

**Figure 2:**
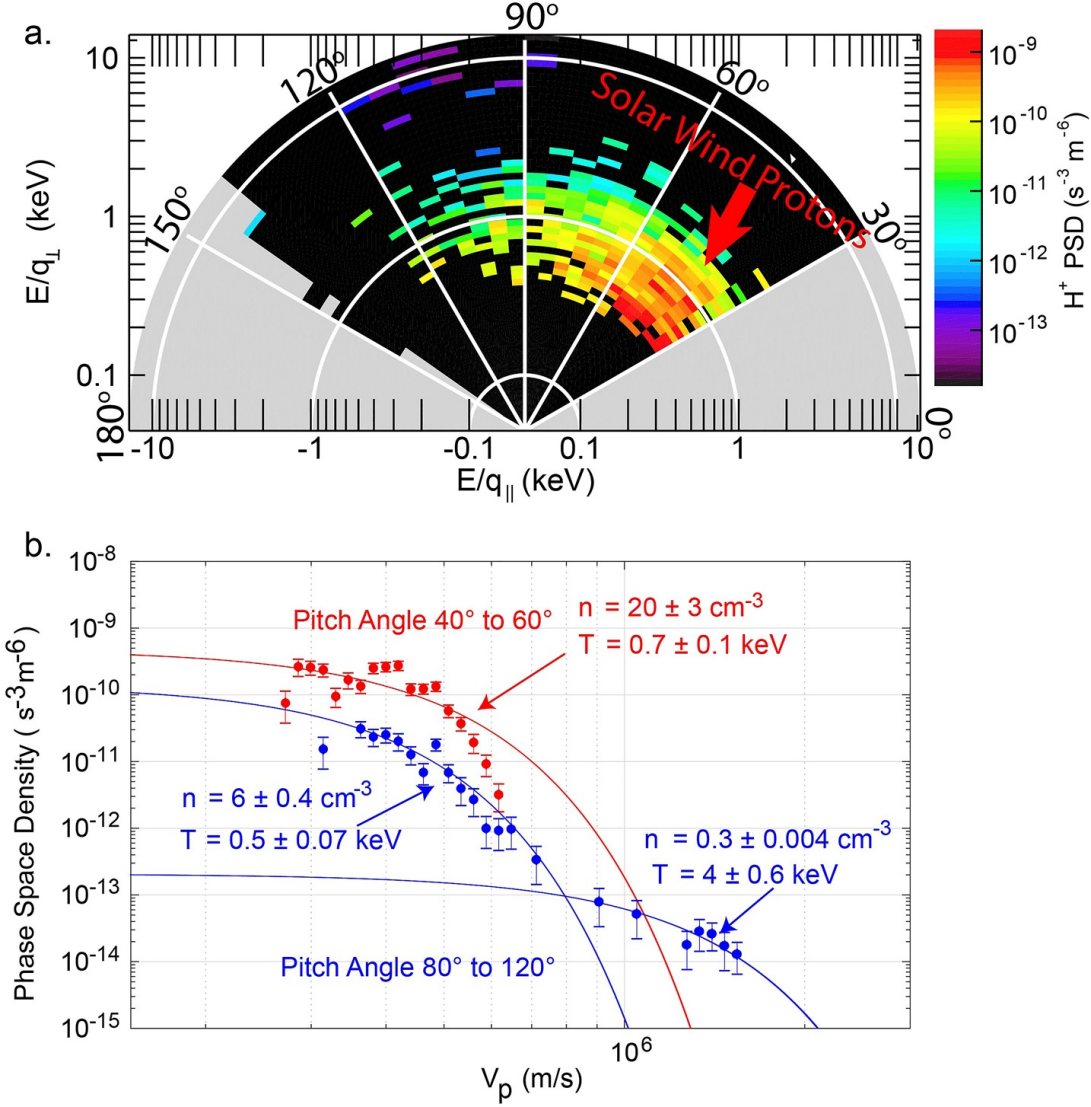
Fast Imaging Plasma Spectrometer (FIPS) measurements of the pitch angle‐energy distribution inside the flux transfer events (FTEs) in the dayside magnetosphere during the MErcury Surface, Space ENvironment, GEochemistry, and Ranging event shown in Figure 1. (a) The distribution of proton phase‐space density (PSD) versus pitch angle. This distribution integrates over all the unwrapped measurements inside the FTEs which the spacecraft crossed inside the magnetosphere. Pitch angle bin size is 5°. Gray regions indicate the pitch angles are out of the field‐of‐view (FOV) of FIPS in the period. (b) Gaussian fits on the protons in the perpendicular direction (pitch angles from 80° to 120°, in blue) and protons in the parallel direction (pitch angles from 40° to 60°, in red). The errorbars of the data points are obtained from the Poisson statistics, which are proportion to $1/\sqrt{counts}$. The counts are the observed counts of each data point. The uncertainties of the fitting results are estimated during the least square curve fit, which correspond to a confident interval of 68.72% (i.e., one standard derivation). Parallel direction contains a cold and dense population. Perpendicular direction contains two populations, one is cold and dense, which is identified to have been injected from the solar wind, and the other is a hot and tenuous magnetospheric population, which could be the quasi‐trapped populations reported by Zhao et al. (2022).

Figure 2a shows the proton phase‐space‐density (PSD) distribution versus pitch angles, which integrates the FIPS measurements inside the FTEs in Figure 1. FIPS provided the proton distribution in pitch angles from 30° to 150° (with more than 40 scans). The cold and dense protons that appeared in the parallel direction (pitch angles smaller than 90°) corresponded to the injected magnetosheath protons along the open magnetic field lines in the FTEs. As shown in Figure 2b, the cold and dense protons with pitch angles from 40° to 60° (the red line) corresponded to a temperature of around 0.7 keV and a density of around 20 cm^−3^, which were consistent with the features of magnetosheath protons. Considering the characteristic energy of 0.7 keV, these high flux solar wind protons would take only around 10 s to travel from the subsolar point to the planet's surface beneath the cusp. These high flux solar wind protons would eventually bombard the planet's surface and cause sputtering.

**Figure 3:**
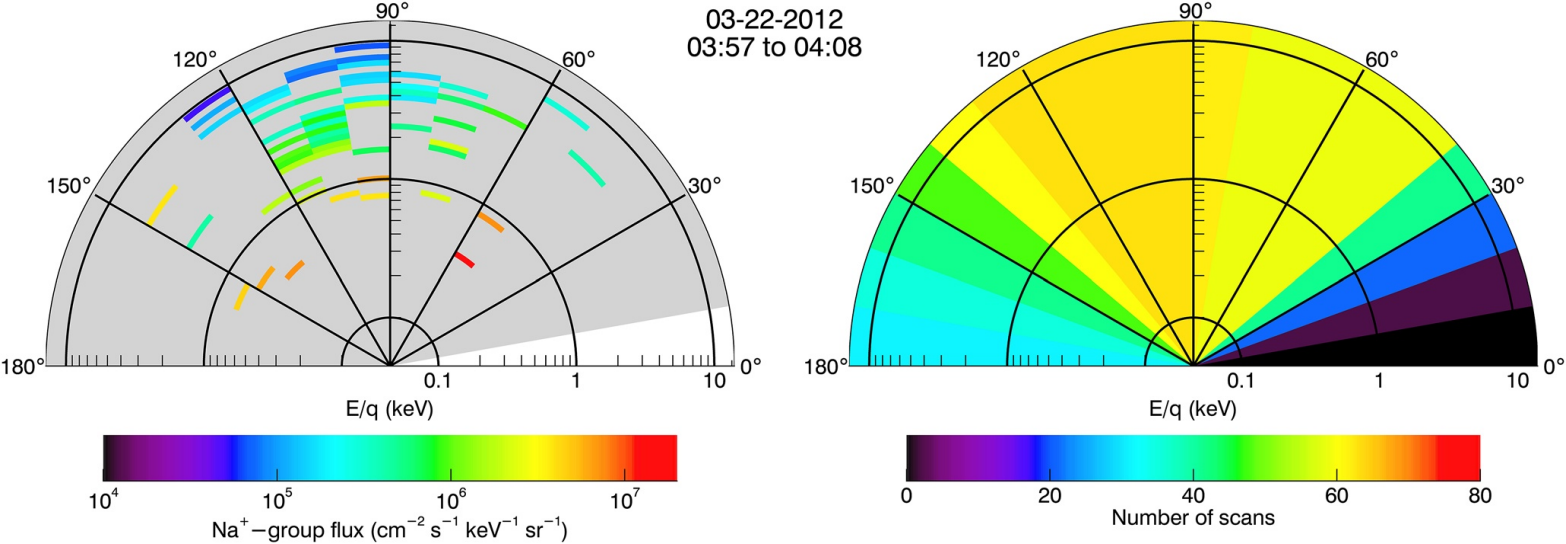
Fast Imaging Plasma Spectrometer (FIPS) measurements of the distribution of the Na^+^‐group ions during the period from 03:57 to 04:08, 22 March 2021 UTC, corresponding to the MErcury Surface, Space ENvironment, GEochemistry, and Ranging event in Figure 1. (a) The distribution of the differential particle flux of Na^+^‐group ions versus pitch angles. (b) The distribution of the number of FIPS scans versus pitch angles. Pitch angle bin size is 10°.

Figure 3 shows the particle flux versus pitch angles of the Na^+^‐group ions during the period from 03:57 to 04:08, 22 March 2012 UTC. This figure integrated the measurements of the Na^+^‐group ions in the dayside magnetosphere. Figure 3a shows that more of the Na^+^‐group ions were moving antiplanetward than planetward, which suggested that the planetary ions were generated and outflowing from the planetary surface. We show and discuss the outflowing Na^+^‐group ions in the next section about statistical analysis.

## 4 Statistical Analysis

### 4.1 Spatial Distribution of Na^+^‐Group Ions and Magnetic Field Line

In the entire mission, MESSENGER traversed the dayside magnetopause around 3,748 times, in which 1953 (about 52%) were accompanied by FTE showers (≥10 FTEs during the magnetopause crossing) (Sun, Slavin, Smith, et al., 2020). Intervals, when MESSENGER crossed the dayside magnetosphere (normally around 15 min) where FTE showers were observed, were defined as FTE shower intervals (1953 events). The remaining dayside magnetosphere crossings (1,795 events) without FTE showers were defined as non‐FTE shower intervals. It is hard to know the magnetopause reconnection conditions in real time with only MESSENGER observations. A study focusing on IMF near Mercury's orbit shows that the IMF is likely to retain a similar state for 10–20 min (James et al., 2017). Therefore, using the FTEs as an indicator of magnetopause reconnection for our study is appropriate.

**Figure 4:**
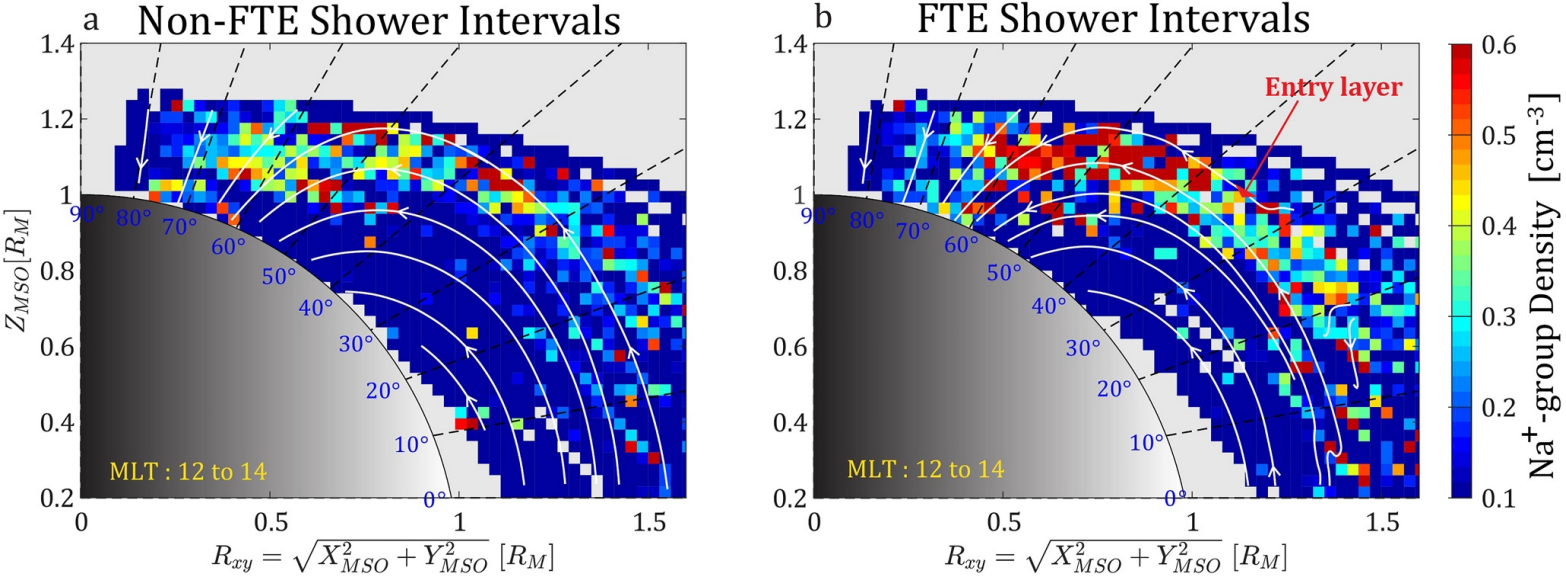
The magnetic field topology as well as MErcury Surface, Space ENvironment, GEochemistry, and Ranging's (MESSENGER's) spatial distribution measurements of the sodium‐group (Na^+^‐group) ions during (a) intervals without flux transfer event (FTE) showers and (b) intervals with FTE showers, shown in the *R*_xy_‐*Z* plane (*R*_xy_ =  $\sqrt{X_{aMSO}^{2}+Y_{aMSO}^{2}}$). Colors indicate the observed density of the Na^+^‐group ions. The white lines represent the magnetic field lines obtained through the average magnetic fields measured by MESSENGER during the intervals without FTE showers and with FTE showers, respectively (see Appendix B on how the field lines are derived). The solar wind entry layer (indicated by the red arrow in b) is determined from the magnetic field topologies during the intervals of FTE showers. In this figure, the measurements of the Na^+^‐group ions were limited in the magnetic local time (MLT) from 12:00 to 14:00.

Figure 4 shows the spatial distributions of the observed density (*n*_obs_) of the Na^+^‐group ions near the noon‐midnight meridian plane (*R*_xy_‐*Z*) plane during the non‐FTE shower intervals (Figure 4a) and the FTE shower intervals (Figure 4b), which are overlaid with the magnetic field lines (in white) during their intervals, respectively. Appendix B describes the derivation of the magnetic field lines. The Na^+^‐group ion density was significantly enhanced during the FTE shower intervals compared to the non‐FTE shower intervals, and the enhancement was concentrated on the newly opened magnetic field lines, which formed a solar wind entry layer at the equatorward boundary of the northern cusp. The density of Na^+^‐group ions in the entry layer was approximately 0.6 cm^−3^ or higher (Figure 4b), while the density was ≲0.3 cm^−3^ in the similar northern cusp region during the non‐FTE showers (Figure 4a). The magnetic field topology during the non‐FTE shower was smooth without signatures of magnetic reconnection.

**Figure 5:**
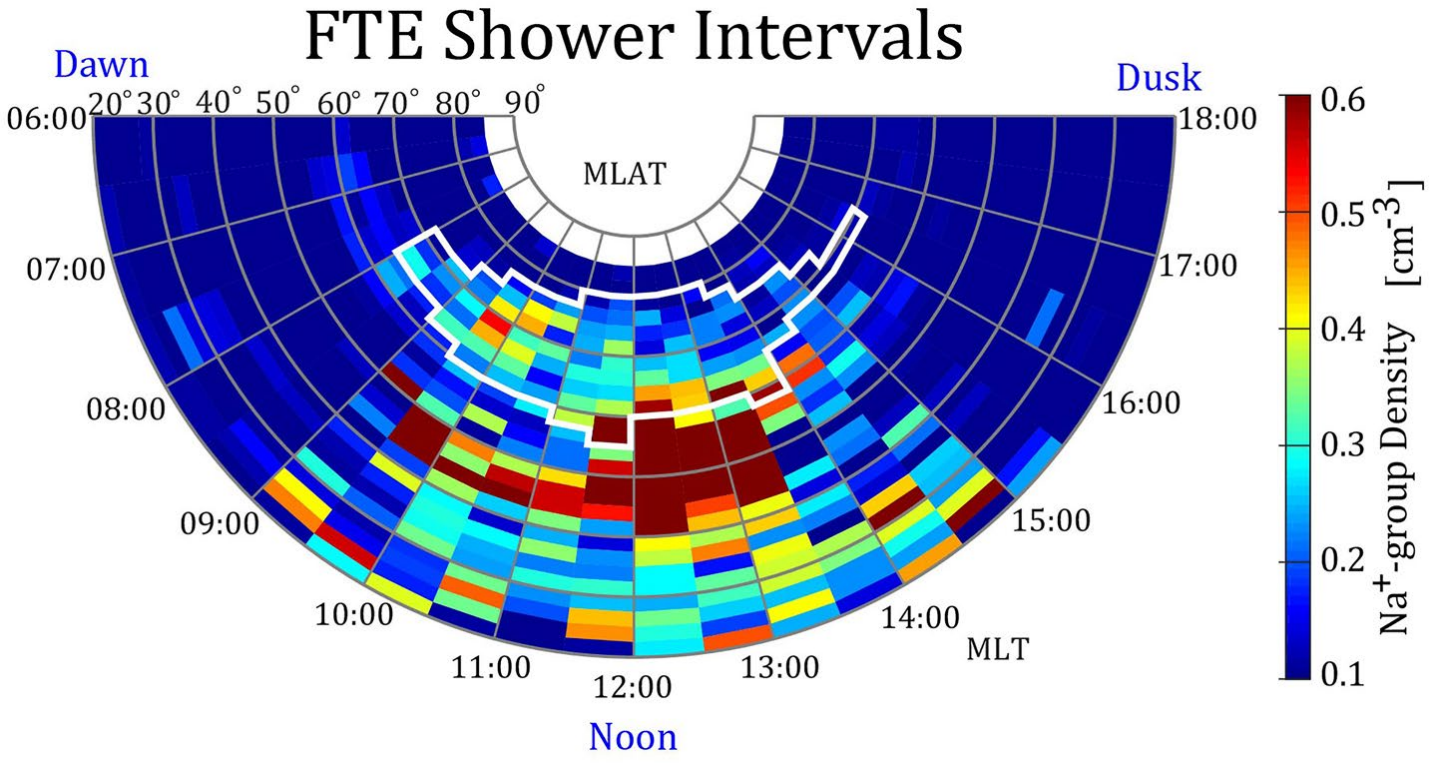
Spatial distributions of sodium‐group (Na^+^‐group) ions during intervals of flux transfer event (FTE) showers in the entry layer along with Mercury's magnetic local time (MLT) and magnetic latitude (MLAT). Colors indicate the observed density of the Na^+^‐group ions. The intervals of FTE showers contain 1953 magnetopause crossings. The white contour includes the cusp which is determined from the spatial distributions of Alpha ions (He^++^, see Figure C1).

Figure 5 shows the distributions of Na^+^‐group ions during FTE shower intervals along with the magnetic local time (MLT) and magnetic latitude (MLAT) on the dayside magnetosphere. The figure shows that Na^+^‐group ions were generally found at an MLT of 09:00–15:00 and MLAT from 20° to 75°. The Na^+^‐group ions were concentrated in MLAT from 40° to 65° and MLT from 12:00 to 13:30 in the postnoon sector and were concentrated in MLAT from 40° to 50° and MLT from 9:30 to 12:00 in the prenoon sector.

### 4.2 Solar Wind Entry Layer

**Figure 6:**
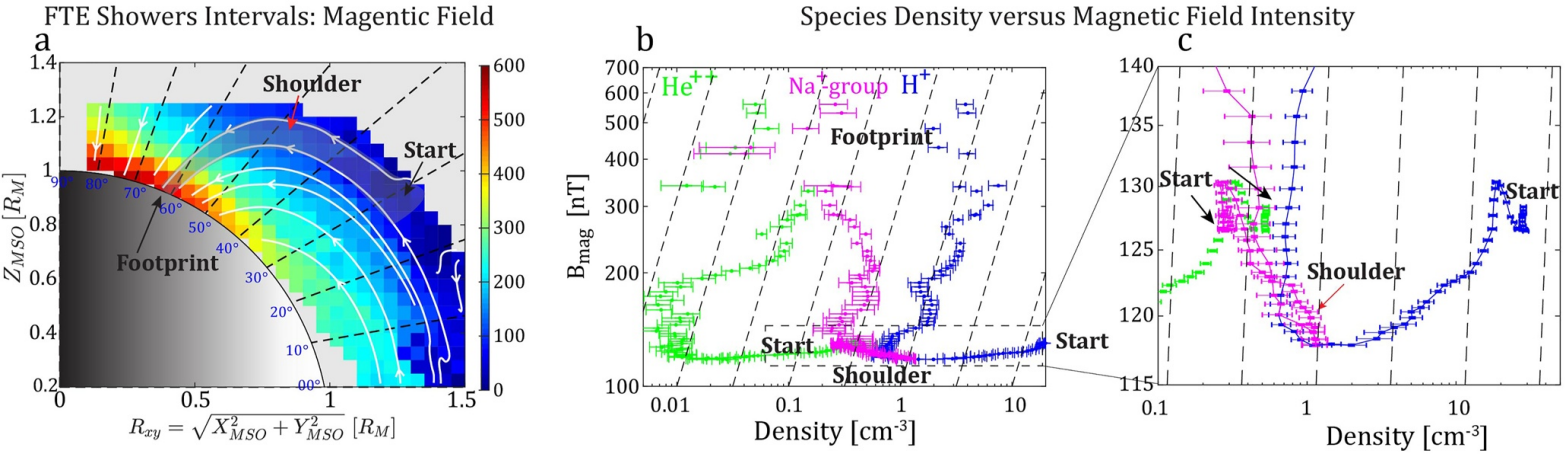
The densities of sodium‐group (Na^+^‐group) ions, protons (H^+^), and alpha ions (He^++^) in the solar wind entry layer on the equatorward boundary of the northern cusp during the intervals of flux transfer event (FTE) showers. (a) the distribution of the averaged magnetic field intensity and the magnetic field lines during FTE shower intervals (the magnetic field lines are the same as shown in Figure 4b). The “start,” “shoulder,” and “footprint” of the entry layer are marked. (b, c) The variations of the observed densities of Na^+^‐group ions (in magenta), H^+^ (in blue), and He^++^ (in green) in the entry layer along with the magnetic field intensity (*B*_mag_). The magnetic field density is the averaged magnetic field intensity measured by MErcury Surface, Space ENvironment, GEochemistry, and Ranging in the entry layer. The dashed lines represent the linear correlation between *n*_density_ and *B*_mag_, that is, *n*_density_/*B*_mag_ = constant. The linear correlation suggests adiabatic travel of ions. In this figure, the measurements of the Na^+^‐group ions, H^+^, and He^++^ were limited in the magnetic local time (MLT) from 10:00 to 14:00.

The FTE shower opened a solar wind entry layer, in which the solar wind particles were channeled into the magnetosphere, impacting the planet's surface and causing sputtering. Figure 6 traces the open magnetic field lines in the entry layer (see Appendix B on how to trace field lines) and shows the densities of H^+^, Na^+^‐group ions, and He^++^ as a function of the magnetic field intensities (*B*_mag_). The densities of H^+^ (around 30 cm^−3^) and He^++^ (around 0.6 cm^−3^) at the “start” of the entry layer were high and decreased until the “shoulder” of the entry layer, where the density was about 1 cm^−3^ for H^+^ and about 0.01 cm^−3^ for He^++^. After the “shoulder,” the densities of H^+^ and He^++^ increased almost linearly with the *B*_mag_ till reaching the “footprint” of the entry layer. The decrease of H^+^ and He^++^ density from the “start” to the “shoulder” implied that most of the solar wind ions transferred poleward, which would form the plasma mantle (DiBraccio et al., 2015; Jasinski et al., 2017; Sun, Slavin, Dewey, et al., 2020). The densities of H^+^ and He^++^ were linearly correlated with the *B*_mag_ suggesting that these solar wind ions adiabatically moved along the flux tubes after passing the “shoulder,” *n*_density_/*B*_mag_ = constant, that is, the plasma content was conserved along the same flux tube, and the densities of H^+^ and He^++^ near the surface were approximately 5 cm^−3^ and 0.2 cm^−3^, respectively. As discussed above, since the energy of the protons was around 1 keV, it would take around 10 s for the protons with a pitch angle of 45° to travel from the subsolar magnetopause to the surface underneath the cusp (about 4,000 km). Therefore, the solar wind entry layer was rapidly formed in less than a minute. The precipitation rates for H^+^ and He^++^ can be estimated to be 6 × 10^12^ and 4 × 10^11^ m^−2^ s^−1^. Meanwhile, the surface area of the cusp was determined to be approximately 3 × 10^12^ m^2^ (see Appendix C on how the surface area of the cusp is determined), the total precipitation rate was estimated to be 2 × 10^25^ s^−1^ for H^+^, and is 1 × 10^24^ s^−1^ for He^++^.

The precipitation rate of H^+^ was around an order of magnitude higher than the average precipitation rate over the cusp obtained by Winslow et al. (2012), which confirmed that intense sputtering occurred during FTE shower intervals at Mercury. The precipitation rate of He^++^ was around an order of magnitude lower than that of the H^+^, but the He^++^ ions could play a significant role in solar wind sputtering (see Szabo et al., 2018). MESSENGER did not provide measurements of low‐energy electrons, however, electrons should be precipitated simultaneously at a similar or even higher rate than the H^+^.

### 4.3 Outflowing Na^+^‐Group Ions

**Figure 7:**
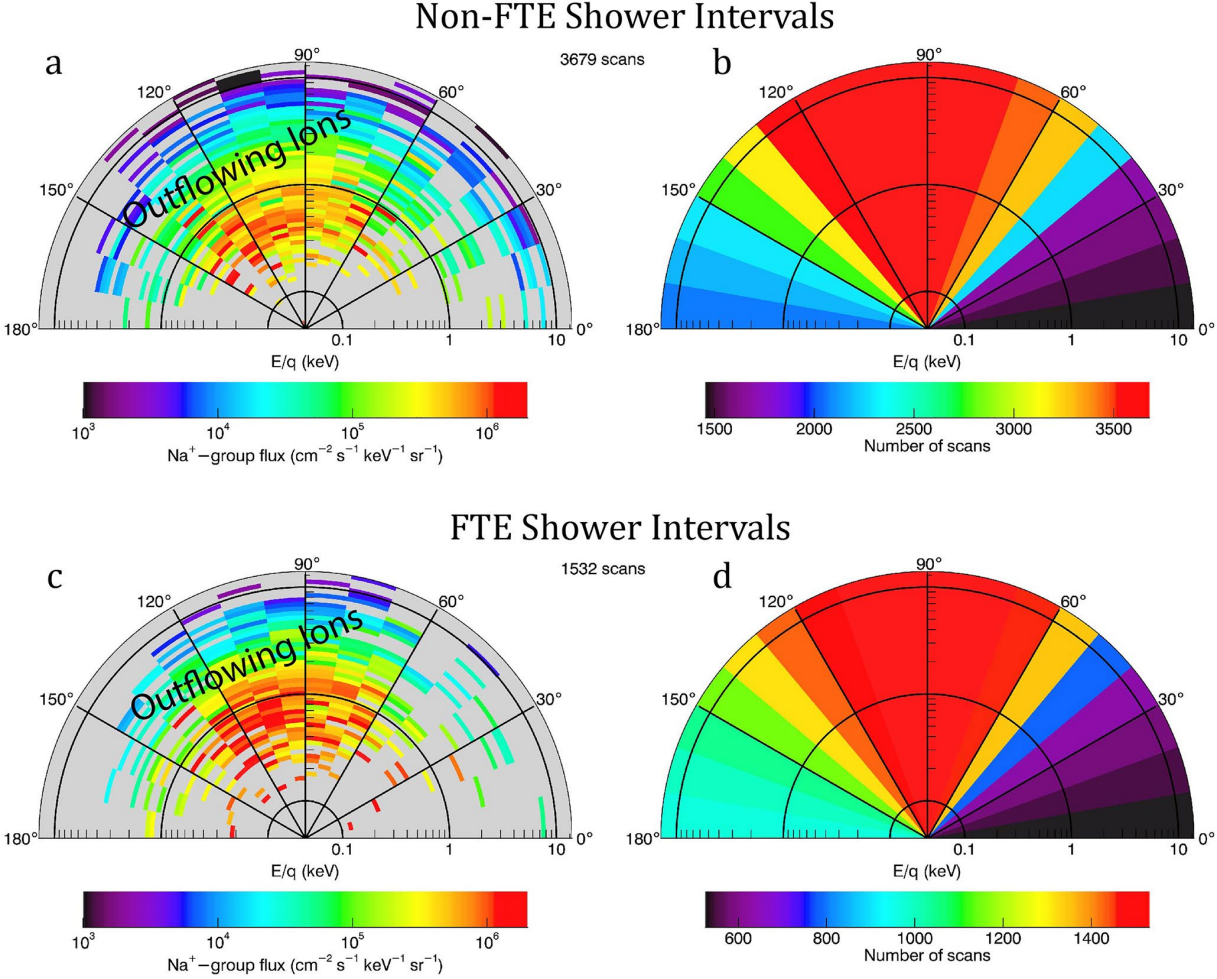
Fast Imaging Plasma Spectrometer (FIPS) measurements of the pitch angle‐energy distribution of the Na^+^‐group ions near the planet's surface beneath the northern cusp. The integration areas include magnetic local time (MLT) from 09:00 to 15:00, magnetic latitude (MLAT) from 55° to 70°, and altitude from 0 to 244 km (0.1 *R*_M_). The pitch angle bin size is 10°. Upper panels (a, b) are for the non‐FTE shower intervals and lower panels (c, d) are for flux transfer event (FTE) shower intervals. Panels on the left (a, c) show the distributions of the differential particle flux (cm^−2^ s^−1^ keV^−1^ sr^−1^). Panels on the right (b, d) show the distributions of the number of scans made by FIPS in each pitch angle bin.

Figure 7 shows the Na^+^‐group ions pitch angle‐energy distributions near the planet's surface beneath the northern cusp for the non‐FTE shower intervals (upper panels) and FTE shower intervals (lower panels). The distribution in Figure 7 integrates over the FIPS measurements in the MLT from 09:00 to 15:00, MLAT from 55° to 70°, and altitude from 0 to 244 km (0.1 *R*_M_). In Figures 7a and 7c, most of the ions were concentrated between pitch angles from 60° to 150°. The distributions of scan numbers of FIPS were shown in Figures 7b and 7d, which showed that FIPS generally covered all pitch angles relative to the magnetic field with the accumulation of the measurements from many MESSENGER's orbits.

The outflowing ions were measured with a range of energies (up to a few keV) but have the highest fluxes at relatively low energies (<400 eV). Table 1 shows the fluxes of Na^+^‐group ions measured at different pitch angles obtained from Figures 7a and 7c. We have integrated the fluxes for Na^+^‐group ions with pitch angles from 60° to 150° and obtained particle fluxes of 7.4 × 10^10^ and 1.1 × 10^11^ m^−2^ s^−1^ for non‐FTE shower intervals and FTE shower intervals, respectively. The flux of Na^+^‐group ions was enhanced around 50% during FTE shower intervals.

**Table 1:** The Flux of Na^+^‐group Ions Near Mercury's Surface Underneath the Cusp, Which Corresponds to the Pitch Angle‐Energy Distribution in Figure 7

Pitch angle (°)	FTE shower intervals		Non‐FTE shower intervals	
	Flux (cm^−2^ s^−1^ sr^−1^)	Number of counts	Flux (cm^−2^ s^−1^ sr^−1^)	Number of counts
0–10	3.9 × 10^4^	1	1.3 × 10^5^	4
10–20	9.1 × 10^4^	2	7.0 × 10^4^	9
20–30	2.5 × 10^5^	60	1.7 × 10^5^	25
30–40	1.7 × 10^5^	5	1.4 × 10^5^	18
40–50	1.5 × 10^5^	10	2.0 × 10^5^	26
50–60	3.3 × 10^5^	15	6.0 × 10^5^	34
60–70	1.1 × 10^6^	48	6.3 × 10^5^	74
70–80	1.2 × 10^6^	100	8.3 × 10^5^	159
80–90	1.4 × 10^6^	137	8.8 × 10^5^	197
90–100	1.5 × 10^6^	176	9.5 × 10^5^	242
100–110	1.7 × 10^6^	179	9.9 × 10^5^	250
110–120	1.6 × 10^6^	138	9.4 × 10^5^	186
120–130	1.2 × 10^6^	77	9.0 × 10^5^	128
130–140	1.1 × 10^6^	33	8.6 × 10^5^	62
140–150	2.9 × 10^5^	21	6.3 × 10^5^	37
150–160	1.8 × 10^5^	14	9.6 × 10^4^	18
160–170	8.1 × 10^4^	7	6.5 × 10^4^	11
170–180	1.1 × 10^5^	3	2.4 × 10^4^	2

## 5 Discussion

### 5.1 Solar Wind Sputtering Corresponds to Outflowing Na^+^‐Group Ions

The enhanced Na^+^‐group ions over the northern cusp were outflowing from the planet's surface. These outflowing Na^+^‐group ions should be fed by ions released from the planet's surface. In this section, we discuss the processes that could release particles out of the planet's surface and try to find out which process generated the outflowing Na^+^‐group ions.

#### 5.1.1 Thermal or Photon‐Stimulated Desorption, and Micrometeoroid Impact Vaporization

Thermal or photon‐stimulated desorption, and micrometeoroid impact vaporization cannot directly produce the enhanced Na^+^‐group ions in the entry layer of the cusp. First, no evidence shows that they can specifically impact the cusp region, instead thermal and photon‐stimulated desorption would generate an exospheric peak at the subsolar of the surface (Domingue et al., 2014; Killen et al., 2007). Micrometeoroid impact vaporization would be higher near the apex of Mercury's orbit (on the dawnside hemisphere) and other sporadic micrometeoroid impact vaporizations are expected to randomly impact the planet's surface, which is unlikely distributed simultaneously as double peaks at northern and southern hemispheres (Pokorný et al., 2018). Second, the processes of thermal and photon‐stimulated desorptions correspond to long‐term variations (days), that is, their timescales are much longer than the 1‐min response time during FTE showers. Third, although micrometeoroid impact vaporization can cause a response on the timescale similar to the FTE showers (Mangano et al., 2007), they should not be correlated with the magnetospheric activity that is caused by FTE showers.

#### 5.1.2 Electron‐Stimulated Desorption and Electron Impact Ionization

The solar wind contains electrons of high fluxes, which is higher than the values of proton fluxes. Electron‐stimulated desorption is another important source for releasing Na neutral (McLain et al., 2011; Yakshinskiy & Madey, 2000), and Na^+^ and potassium ions (K^+^) (McLain et al., 2011). Ions resulting from the electron‐stimulated desorption are unlikely to have high velocities or large gyro‐radii and would therefore remain attach to the planet's surface, which might enhance the effectiveness of the ion sputtering.

**Figure 8:**
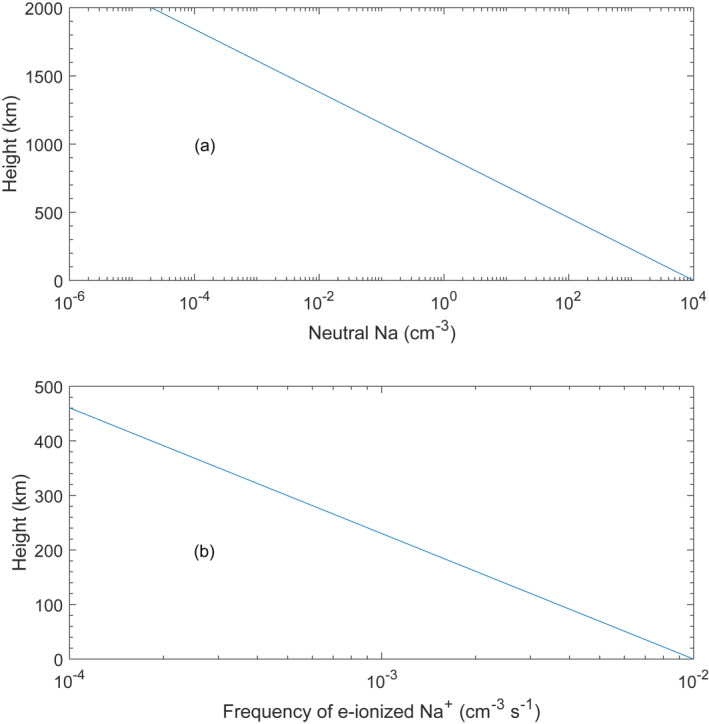
Estimation of electron impact ionization of neutral Na. Upper panel (a): the density profile of neutral Na along with height, that is, altitude; Bottom panel (b): the production rate of Na^+^ ions due to electron impact ionization along with height. The surface density of the neutral Na is 1 × 10^4^ cm^−3^ with a scale height of 100 km. The electron temperature is set to be 100 eV. The neutral Na with the density of 1 × 10^4^ cm^−3^ corresponds to the upper value of neutral Na near the terminator of Mercury.

The electron impact ionization can contribute to the ionizations of neutrals in the atmosphere of Comets (Cravens et al., 1987), Venus and Mars (Ramstad & Barabash, 2021). In Figure 8, we make an estimation of electron ionization of neutral Na near Mercury's surface in the solar wind entry layer. The solar wind electron density is assumed to be comparable to the solar wind proton number density, *n*_e_ ∼ 30 cm^−3^, and the electron temperature is assumed to be 100 eV. The ionization frequency due to electrons can be calculated from

$$R_{ioni}=n_{e}\int_{v_{pot}}^{\infty }v_{e}\sigma_{s}\left(v_{e}\right)f\left(v_{e}\right)4\pi v_{e}^{2}dv_{e}$$

(*Cravens* et al., 1987). The $v_{pot}$is the velocity corresponding to the ionization potential of Na (around 5 eV); $\sigma_{s}$is the electron ionization cross section of Na, which is a function of the electron velocity (*v*_e_). Here we employ the $\sigma_{s}$from Lotz (1967). The ionization frequency is estimated to be 1 × 10^−6^ s^−1^.

Considering a surface density of 1 × 10^4^ cm^−3^ and the scale height of 100 km for the neutral Na (Cassidy et al., 2015) (Figure 8a), the production rate of Na^+^ at the altitude of ∼100 km is estimated to be ∼2 × 10^−3^ cm^−3^ s^−1^ (Figure 8b). The surface density of neutral Na in Figure 8a corresponds to the upper value of neutral Na near the terminator of Mercury. The scale height corresponds to an average value (see Cassidy et al., 2015).

If the bombarding from the solar wind electrons lasts around 3 s, the ionized Na^+^ can have a density of approximately 0.01 cm^−3^. The cusp filament lasts around 3 s on average (Poh et al., 2016), which corresponds to the bombarding associated with solar wind precipitation. The observed density of Na^+^ was 0.6 cm^−3^, which indicates that the density of Na^+^ was around 2 cm^−3^ (0.6 cm^−3^ × 4π/1.15 π). Therefore, we conclude that electron ionization contributes less than 1% of the Na^+^ observed in the solar wind entry layer.

#### 5.1.3 Solar Wind Ions Sputtering

In Figure 6, from the “start” to the “shoulder,” the densities of solar wind H^+^ and He^++^ decreased indicating that they convected poleward. However, the enhancement of the Na^+^‐group ions suggested the Na^+^‐group ions were piled up in the entry layer from the start to the shoulder, which indicated that the Na^+^‐group ions were continuously generated and outflowing from the planet's surface. Those outflowing Na^+^‐group ions in Figure 7 were close to the planet's surface with an altitude from 0 to 244 km. The sputtering of solar wind energetic ions can release atoms/ions from the planet's regolith with energies up to a few hundreds eV (Hofer, 1991; Sieveka & Johnson, 1984; Sigmund, 1969; Mura et al., 2007). However, only a small fraction of atoms were in the energy higher than 10 eV, which could not account for the high particle flux of a few hundred eV to a few keV of the Na^+^‐group ions observed in Figure 7.

Another feature was that the flux of the Na^+^‐group ions was enhanced (>50%) during FTE shower intervals, which was another evidence that they were generated by the solar wind sputtering. Considering the precipitation rate of H^+^ is 6 × 10^12^ m^−2^ s^−1^ and the outflowing particle flux of the Na^+^‐group ions is 1 × 10^11^ m^−2^ s^−1^ during FTE shower intervals, the yield for the sputtered Na^+^‐group ions can be calculated to be approximately 2%. Those sputtered ions would be first tied to the magnetic field line in the solar wind entry layer by the Lorentz force. If they could reach high enough altitudes, they would then be accelerated by the convection electric field in the entry layer, which was driven by the potential drop associated with the dayside magnetopause reconnection since the magnetic field lines in the entry layer were newly opened by the magnetopause reconnection. This process could be similar to the pickup process of Na^+^ test particles shown in Glass et al. (2021). Those authors suggested that the large‐scale magnetospheric convection electric field energized Na^+^ test particles from very low altitudes on the dayside enough that they could gyrate through the magnetopause. The Na^+^ ion does not need very high velocity to reach the observed a few hundred eV to keV energy. The 1 keV Na^+^ ion corresponds to a speed of ∼90 km/s.

The potential differences between the Mercury's surface and MESSENGER's FIPS might provide initial energy for the Na^+^ ion, enough to reach the convection electric field of the entry layer. We do not know the potential for either of them but they should be both positively charged since the photoelectron current would be a major current source. The Japanese lunar orbiter SELENE (Kaguya) provide such measurement of Moon‐originating ions when SELENE was located in Earth's magnetosphere (Saito et al., 2010; Tanaka et al., 2009). However, the exact acceleration mechanism for these observed a few hundred eV to keV Na^+^‐group ions near Mercury's surface is still unknown.

When the sputtered ions moving antiplanetward along the magnetic field lines, that is, outflowing, reach higher altitudes, they would be subject to centrifugal acceleration (Delcourt et al., 2012) and they would also encounter the solar wind convection electric field and be accelerated further (also see Glass et al., 2021; Raines et al., 2014). Once in the magnetosheath, they would move along the magnetopause and reach keV energies before passing through the cusp (at similar altitudes to our observations in Figures 1b and 3).

Note that the Na^+^‐group ions were less dense but did not disappear during the non‐FTE shower intervals. These could be because (a) the solar wind sputtering did not completely disappear as the cusp existed during both FTE shower intervals and non‐FTE shower intervals; (b) those photoionized ions were accelerated by the solar wind and transferred into the magnetosphere (Jasinski, Cassidy, et al., 2021; Raines et al., 2014; Sarantos et al., 2009; Wurz et al., 2019).

### 5.2 Ion Escape Channel at Mercury

The integration over the entry layer with MLT from 06:00 to 18:00 gave a Na^+^‐group ions content of around 1 × 10^25^. Considering a convection speed of 200 km/s and a scale of 1.36 *R*_M_ of the entry layer, the average transport rate of Na^+^‐group ions from the dayside to the nightside can be estimated to be around 6 × 10^23^ s^−1^. Note that (a) we used the Alfvén speed at the start of the entry layer (approximately 230 km/s) to be the approximate convection speed; (b) this transport rate only considered the Na^+^‐group ions in the entry layer in the northern hemisphere. The whole transport rate considering both northern and southern hemispheres should be double the 6 × 10^23^ s^−1^ and was approximately 1 × 10^24^ s^−1^.

The transport rate of the Na^+^‐group ions (1 × 10^24^ s^−1^) from the dayside to the nightside is comparable to the neutral Na escape rate (0.5–1.3 × 10^24^ s^−1^) in the Na tail (Schmidt et al., 2010), which is primarily caused by the radiation‐pressure‐induced acceleration (Ip, 1986) and therefore is highly variable along Mercury's year. However, it is not clear what proportion of the estimated Na^+^‐group ions transport rate is due to Na^+^ ion. In the northern hemisphere of Mercury, the surface chemical composition (in wt %) for Na is 5.74%, Mg is 7.55%, Al is 6.04%, Si is 30.19% (McCoy et al., 2018). The neutral Na, therefore, forms around 11% of all four of these species at the surface. If we assumed that Na^+^ ion was present in the same proportion as the surface composition of the Na^+^‐group species, then the transport rate of Na^+^ ion was around 1 × 10^23^ s^−1^, which was several times lower than the escape rate of neutral Na in the Na tail. We note that there was no evidence that the Na^+^ ion was in the same proportion as the neutral Na at the surface. A lack of in situ/laboratory experiments in this area meant we had to make this simple assumption.

The transport of Na^+^‐group ions during the FTE showers was likely an escape channel for Mercury's planetary ions, which was driven by the solar wind‐magnetosphere‐surface coupling process and was different from the constant exospheric sodium loss due to the photoionization of the sodium exosphere. Photoionization removes approximately 0.9–4 × 10^24^ ions/s of Na^+^‐group ions from the exosphere, with variations driven by seasons (Jasinski, Cassidy, et al., 2021). Our study focuses on short (minutes) timescales of the high latitude regions, while photoionization produces long‐term (seasonal) variation of the global exosphere. Ion escape is observed in the inner planets of our solar system, that is, Mercury, Venus, Earth and Mars. The O^+^ ions escape from the dayside polar cap region at Earth at rates of 10^24^ to 10^26^ s^−1^ (Slapak et al., 2017, 2018). The energetic ion plume of escaping O^+^ ions observed in the induced magnetospheres at Mars is at the rate of 10^24^ to 10^25^ s^−1^ (Lundin et al., 2013) and Venus at approximately 10^25^ s^−1^ (McComas et al., 1986). For those planets without a global intrinsic magnetic field, that is, Mars and Venus, the escape ions are ionized by the solar ultraviolet (UV), electron impact ionization, or due to charge exchanges, which forms a constant escape channel (Dubinin et al., 2011; Ramstad & Barabash, 2021). The escape of O^+^ at Earth depends on the solar wind parameters (Schillings et al., 2019; Slapak et al., 2017), which is similar to the escape channel of Na^+^‐group ions found in this study.

### 5.3 Influence on Neutral Exosphere

In this section, we discuss how the solar wind sputtering influences the neutral Na exosphere at Mercury. At first, we estimate the surface density of neutral Na by considering the surface release flux of the Na^+^‐group ions.

If we assumed that the Na^+^ ions formed 11% of the Na^+^‐group ions that were released from the surface by solar wind particles during FTE shower intervals (Figure 7c and Table 1), similar to the previous assumption in Section 5.2, then the release flux of Na^+^ ions in the northern hemisphere was around 1 × 10^10^ m^−2^ s^−1^. Since the sputtered Na^+^ ions account for only 5–10% of the sputtered atoms (Benninghoven, 1975; Hofer, 1991), the sputtered neutral Na would approximately be an order of magnitude higher, which is around 1 × 10^11^ m^−2^ s^−1^. The exospheric density of the neutral Na at the surface (*n*_surf_) can be estimated from

$$n_{\text{surf}}=f_{\text{Na}}/v_{\text{release}},\;$$

where *v*_release_ is the release velocity of neutral Na. The sputtering energy spectrum peaks at around one eV. Thus, we consider *v*_release_ to be 3 km/s on average. As a result, *n*_surf_ ∼ 3 × 10^7^ m^−3^.

The exospheric surface densities of neutral Na range from 10^9^ to 10^11^ atoms/m^3^ near Mercury's subsolar point and from 10^8^ to 10^10^ atoms/m^3^ near the terminator (Cassidy et al., 2015). Hence, the FTE shower, on average, could likely enhance a considerable portion (≳10%) of the neutral Na in the cusp region through sputtering in minutes, which can likely cause the short‐term variations of the Na emissions observed by ground‐based telescope (Massetti et al., 2017; Orsini et al., 2018). Our study provides clear evidence that dayside magnetopause reconnection, specifically FTE showers, injects solar wind ions into the cusps and enhance the Na^+^‐group ions in the high latitude magnetosphere. However, the causes of the short‐term variability of the neutral Na exosphere could be more complex. This study provides a candidate, specifically FTE showers, for causing the short‐term variations, which does not exclude that other processes might additionally cause short‐term variations of the neutral Na exosphere.

Second, we can obtain the surface density of neutral Na by considering the estimated impact of solar wind proton fluxes and the known parameters derived for sputtering from the analytical model or laboratory. In the exospheric circulation models (Mura et al., 2007; Orsini et al., 2021), similar solar wind impact flux (10^13^ m^−2 ^s^−1^) can produce an exospheric surface density of neutral Na of 10^8^ m^−3^. Moreover, we can employ the sputtering yield derived from laboratory experiments. The estimated solar wind proton impact flux is *f*_impact_ ∼ 1 × 10^13^ m^−2^ s^−1^. The sputtered neutral Na,

$$F_{\text{Na}}=f_{\text{impact}}\times y\times C,\;$$

where *y* is the yield (number of atoms released for each impacting ion) with a maximum measured value of 0.08 (Lammer et al., 2003; Johnson & Baragiola, 1991) and *C* is the relative surface composition for Na, which at maximum is 0.06 (Peplowski et al., 2014). Hence, the maximum *F*_Na_ is around 5 × 10^10^ m^−2^ s^−1^. Then, dividing by the *v*_release_, the *n*_surf_ is estimated to be approximately 2 × 10^7^ m^−3^.

There are differences between the models and the estimation based on the sputtered Na^+^. The *n*_surf_ (3 × 10^7^ m^−3^) in this study, which is estimated from the sputtered Na^+^‐group ions, is more close to the *n*_surf_ (2 × 10^7^ m^−3^) estimated from the sputtering yield derived from laboratory experiments, but they are several times smaller than the value (10^8^ m^−3^) obtained from the exospheric circulation models. The differences are not large and are within an order of magnitude. These differences could be due to several processes that have not been studied. For example, (a), the production rate of neutral Na depends on several factors, including temperature, the composition of the surface, and mineralogy (Killen et al., 2007). Weider et al. (2015) provided the global mapping of major elements on the surface of Mercury. However, the mineralogy about surface bounds still has not been well derived; (b) the solar wind includes alpha ions (He^++^) with a precipitation rate of around 1 × 10^24^ s^−1^. The He^++^ ions could enhance the yield of the sputtering (Szabo et al., 2018). The solar wind also includes a large portion of electrons. The electron‐stimulated desorption could also affect the release of neutrals and ions from the planet's surface.

## 6 Further Impact and Future Mission

The results from this study can influence a variety of aspects. Not only is solar wind‐magnetopause reconnection important for directly influencing the exospheric dynamics and planetary ion escape at Mercury, but also magnetic reconnection can input explosive energy from the solar or a stellar wind into the magnetosphere of planets or an exoplanet under intense external driving (Barclay et al., 2013) similar to Mercury. For example, Ganymede, one of the Galilean moons, has a global magnetic field (Kivelson et al., 1996) and is located in a sub‐Alfvénic corotation flow in Jupiter's magnetosphere. The sub‐Alfvénic flow refers to flow speed smaller than the background Alfvén speed, and therefore corresponds to low *M*_A_. In a recent simulation study, Zhou et al. (2019, 2020) show that magnetic reconnection can frequently generate magnetic flux ropes on the magnetopause and input a significant amount of energy into Ganymede's magnetosphere. Exoplanets with a global magnetic field close to their primary stars could be exposed to similar low *M*_A_ stellar wind (Ip et al., 2004). At those planets, intense magnetic reconnection can be expected to occur that leads to efficient transport of plasma and energy from the stellar wind into the planet's atmosphere or surface, which can facilitate atmospheric escape, as simulated by Egan et al. (2019), and therefore affect the habitability of planets and exoplanets.

A joint European Space Agency (ESA)‐Japan Aerospace Exploration Agency (JAXA) mission, BepiColombo (Milillo et al., 2020), consisting of the Mercury Planetary Orbiter (MPO) and the Mercury Magnetospheric Orbiter (MMO, or Mio), made its first flyby of Mercury in October 2021 with Mercury orbit insertion scheduled in late 2025 or early 2026. BepiColombo will provide many comprehensive measurements on Mercury's magnetosphere and exosphere, especially those higher resolutions measurements for different ion species, that is, Mercury Plasma Particle Experiment (MPPE) (Saito et al., 2021) onboard Mio, and neutrals, that is, Search for Exospheric Refilling and Emitted Natural Abundances (SERENA) (Orsini et al., 2021). Moreover, MPO and Mio will have much broader altitudinal coverage of both northern and southern cusps than MESSENGER was able to achieve. At times, one spacecraft will serve as a solar wind monitor to the other spacecraft inside the magnetosphere. The impact of magnetopause reconnection on Mercury's exospheric dynamics will be investigated in much detail.

## Figures and Tables

**Figure B1:**
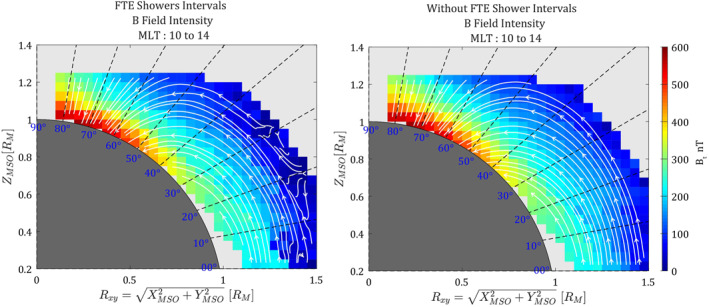
Spatial distributions of magnetic field intensity and the derived magnetic field lines during intervals of flux transfer event (FTE) showers (left) and intervals of non‐FTE showers (right) in the *R*_xy_‐Z plane (*R*_xy_ =  $\sqrt{X_{aMSO}^{2}+Y_{aMSO}^{2}}$). Colors indicate the observed magnetic field intensity from MErcury Surface, Space ENvironment, GEochemistry, and Ranging, which were averaged in the magnetic local time (MLT) from 10:00 to 14:00. The white lines represent the magnetic field lines derived through the averaged magnetic field intensities. The left figure during the intervals of FTE showers, there are few curved magnetic field lines on the outer edge, which should be signatures of magnetic reconnection or FTEs. On the right figure, the magnetic field lines are smooth.

**Figure C1:**
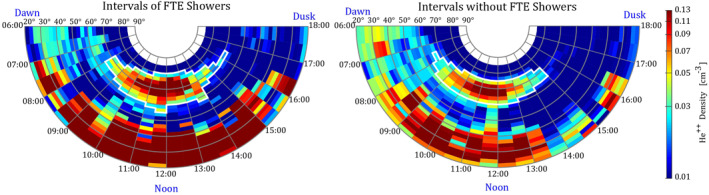
Spatial distributions of Alpha (He^++^) particles during the intervals of FTE showers (left) versus intervals without FTE showers (right) along Mercury's magnetic local time (MLT) and magnetic latitude (MLAT). Colors indicate the observed density of He^++^. The white contours include regions with He^++^ density higher than 0.03 cm^−3^.

## Data Availability

MESSENGER data are available through the Planetary Plasma Interactions (PPI) Node of the NASA Planetary Data System (PDS) at https://pds-ppi.igpp.ucla.edu/. The magnetic field data measured by MAG are available at https://pds-ppi.igpp.ucla.edu/search/view/?f=yes&id=pds://PPI/MESS-E_V_H_SW-MAG-3-CDR-CALIBRATED-V1.0, the ion data measured by FIPS are available at https://pds-ppi.igpp.ucla.edu/search/view/?f=yes&id=pds://PPI/MESS-E_V_H_SW-EPPS-3-FIPS-DDR-V2.0. The list of Mercury's dayside magnetopause crossings (3,748 crossings) made by MESSENGER from 11 March 2011 to 30 April 2015 is available at the supporting information of Sun, Slavin, Smith et al. [2020], https://doi.org/10.1029/2020GL089784. The model data were obtained from simulations using the SWMF/BATSRUS code developed at the University of Michigan, which is publicly available at http://csem.engin.umich.edu/tools/swmf/.

## Appendix A Hall Magnetohydrodynamics (MHD) Simulation

The simulation result is shown in Figure 1 (right panel) and is extracted from a global Hall‐MHD simulation of Mercury's magnetosphere. The simulation is performed using the Hall‐MHD version of the Block‐Adaptive Tree Solarwind Roe‐type Upwind Scheme (BATS‐R‐US) code (Tóth et al., 2012) that enables us to properly simulate fast magnetic reconnection on the magnetopause. In our resistive, Hall‐MHD treatment, the generalized Ohm's law reads as: $\vec{E}=\eta \vec{J}-\vec{u}\times \vec{B}+\frac{1}{ne}\vec{J}\times \vec{B}$, where $\eta \vec{J}$is the resistivity term, $\vec{u}$, *n*, and $\vec{B}$are plasma velocity, density, and magnetic field, respectively. The Hall‐term (the last term on the right‐hand side of the above equation) is important for plasma dynamics on scales shorter than ion inertial scale lengths, but greater than electron inertial scale lengths. The magnetic field lines are frozen to the electron fluid but not to the ion fluid due to the Hall effect. It has been demonstrated that Hall‐MHD appears to be the minimal modification required for an MHD code to reproduce the fast reconnection process seen in particle and hybrid simulations (Birn et al., 2001; Chen et al., 2019). Our global Hall‐MHD model also electromagnetically couples Mercury's interior to the surrounding magnetosphere, allowing us to directly simulate the induction effect arising from Mercury's large‐size conducting core (Jia et al., 2015, 2019). This is achieved primarily through the resistivity term included in the generalized Ohm's law, for which different resistivity (or inversely conductivity) values are prescribed to represent different electrical properties of the planet's mantle and core.

## Appendix B Derivation and Trace of Magnetic Field Lines

The measurements of the magnetic field vector during the FTE shower intervals and the non‐FTE shower intervals are averaged over the dayside magnetosphere, respectively. In Figures 4 and 6, the magnetic field lines are derived from the averaged magnetic field vectors within the local times from 10 to 14 MLT. Figure B1 shows the spatial distributions of magnetic field intensity and the magnetic field lines during intervals of FTE showers and intervals of non‐FTE showers. FTE shower intervals contain 1953 dayside magnetosphere crossings, and non‐FTE shower intervals contain 1795 dayside magnetosphere crossings.

In Figure 6, we have traced the magnetic field lines in the solar wind entry layer, which is the shaded region between the closed and open magnetic field lines as shown on the left panel. The starting point is indicated by the “Start,” and the endpoint the “Footprint.” The magnetic field intensity and the densities of ion species are obtained through average over the entire entry layer within the MLT from 12 to 14 MLT.

## Appendix C Determination of Surface Area of Cusp

The area of the northern cusp is determined from the distribution of the alpha ions (He^++^) measured by FIPS. Figure C1 includes a spatial distribution of the alpha ions during the intervals of FTE showers and non‐FTE‐showers, respectively, along Mercury's magnetic local time (MLT) and magnetic latitude (MLAT). The northern cusp is defined to be the area in the high latitude with the densities of the alpha ions being larger than 0.03 cm^−3^. The area of the cusp is calculated from $r^{2}\times \cos\left(\text{MLAT}\right)\times \Delta \text{MLONG}\times \Delta \text{MLAT}$, where *r* represents the radial length, ∆MLONG is the width of the angle along the magnetic longitude (i.e., the MLT), and ∆MLAT is the width of the angle along the magnetic latitude. The ∆MLAT is not a constant along different MLT. We calculate the value of the area in each grid of MLT and then integrate them. The surface area (*A*) is estimated to be around 3 × 10^12^ m^2^.
